# Apatinib inhibits cellular invasion and migration by fusion kinase KIF5B-RET via suppressing RET/Src signaling pathway

**DOI:** 10.18632/oncotarget.10985

**Published:** 2016-08-01

**Authors:** Chen Lin, Shanshan Wang, Weiwei Xie, Rongliang Zheng, Yu Gan, Jianhua Chang

**Affiliations:** ^1^ Department of Medical Oncology, Fudan University Shanghai Cancer Center, Shanghai, 200032, P.R. China; ^2^ Department of Nuclear Medicine, Sun Yat-Sen University Cancer Center, Guangzhou, 510000, P.R. China; ^3^ State Key Laboratory of Oncogenes and Related Genes, Shanghai Cancer Institute, Renji Hospital, Shanghai Jiao Tong University School of Medicine, Shanghai, 200032, P.R. China

**Keywords:** apatinib, KIF5B-RET, invasion and migration, Src signaling pathway, targeted therapy

## Abstract

The Rearranged during transfection (RET) fusion gene is a newly identified oncogenic mutation in non-small cell lung cancer (NSCLC). The aim of this study is to explore the biological functions of the gene in tumorigenesis and metastasis in RET gene fusion-driven preclinical models. We also investigate the anti-tumor activity of Apatinib, a potent inhibitor of VEGFR-2, PDGFR-β, c-Src and RET, in RET-rearranged lung adenocarcinoma, together with the mechanisms underlying. Our results suggested that KIF5B-RET fusion gene promoted cell invasion and migration, which were probably mediated through Src signaling pathway. Apatinib exerted its anti-cancer effect not only via cytotoxicity, but also via inhibition of migration and invasion by suppressing RET/Src signaling pathway, supporting a potential role for Apatinib in the treatment of KIF5B-RET driven tumors.

## INTRODUCTION

Treatment strategies for non-small cell lung cancer (NSCLC) have evolved to an emphasis on molecularly targeted therapy based on genomic classification of patients [1]. The discovery of EGFR mutations and ALK rearrangements has remarkably revolutionized the therapeutic landscape of NSCLC. Traditional platinum-based chemotherapy is gradually replaced by targeted drugs, like EGFR tyrosine kinase inhibitors (erlotinib, geftinib, afatinib) and ALK inhibitors (crizotinib and ceritinib) even in the front-line setting [2–3]. To date, a series of molecular alterations, such as BRAF, PIK3CA, BRAF, HER2 mutations and ROS1 translocations, have been successively recognized as driver mutations in NSCLC [4]. The patient benefits from antagonizing genetic alterations have been demonstrated in excessive clinical trials, including prolonged survival and improved quality of life. Identification of oncogenic drivers has been highlighted in the era of precision medicine [5].

Between late 2011 and early 2012, KIF5B-RET fusion gene was firstly identified in NSCLC by four independent research groups [6–9]. It is formed by a pericentric inversion on chromosome 10, which constitutes a coiled coil domain and induces autophosphorylation of RET tyrosine kinase, thereby activates uncontrolled signal transduction and leads to tumorigenesis [10–11]. The fusion gene is mutually exclusive to other major mutations like EGFR, KRAS, ALK and BRAF and has been considered as a novel driver mutation in lung adenocarcinomas (LADC) [6–8]. The overall prevalence of RET fusion in lung adenocarcinomas is 1−2% according to existing literatures, and it increases to 6−19% for tumors without other genetic variants [12]. Although *in vitro* and xenograft model data support the tumorigenicity potential of RET chimeras, information about other biological functions, like proliferation, migration and invasion is lacking.

Apatinib, a novel, orally administered receptor tyrosine kinase (RTK) inhibitor, targets the vascular endothelial growth factor receptor-2 (VEGFR-2), RET, platelet-derived growth factor-β (PDGFR-β), v-Src sarcoma viral oncogene homolog (c-Src), and stem cell factor receptor (c-Kit) [13–14]. Highly selective competition within the ATP-binding site of VEGFR-2 in the cell will block the downstream signal transduction and inhibit tumor angiogenesis [15]. Based on this mechanism, phase III clinical trials have been conducted aiming at gastric carcinoma in China and Apatinib has proved to be effective and safe in the treatment of advanced gastric cancer patients [16]. However, anti-tumor activity of Apatinib in RET-rearranged lung cancer has never been reported. Considering the huge population baseline of NSCLC patients worldwide, the treatment for RET fusion-positive NSCLC patients with RET inhibitors has great significance both in theory and in practice.

Herein, we explored the biological functions of the gene in tumorigenesis and metastasis in RET gene fusion-driven preclinical models. The anti-tumor activity of Apatinib was also evaluated to explore the therapeutic potential in RET fusion–driven LADC.

## RESULTS

### Establishment of stable transfected cell lines

**Figure 1:**
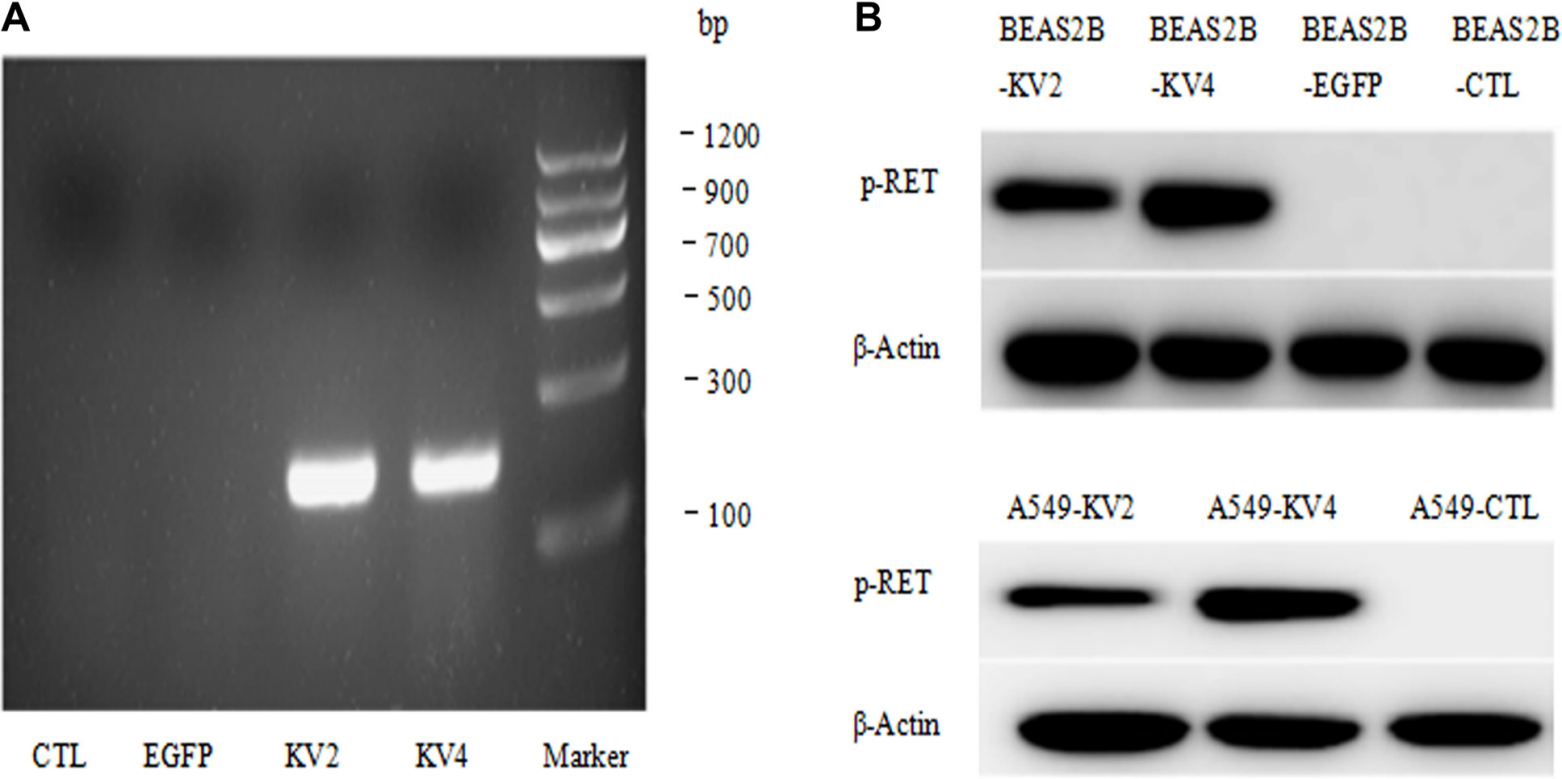
Establishment of stable transfected BEAS-2B and A549cell lines (**A**) The RT-PCR method showed the mRNA over-expression of the fusion gene after transfection. (**B**) The stable transfected cell lines successfully expressed phosphorylated RET, suggesting that KIF5B-RET could automatically activate RET kinase.

KIF5B-RET gene in our research had two isomers, variant 2 and variant 4(mentioned next as KV2 and KV4). The RT-PCR method showed the mRNA over-expression of the fusion gene after transfection (Figure 1A). The stable transfected cell lines successfully expressed phosphorylated RET, suggesting that KIF5B-RET could automatically activate RET kinase (Figure 1B).

### KIF5B-RET fusion gene was capable of inducing malignant transformation

**Figure 2:**
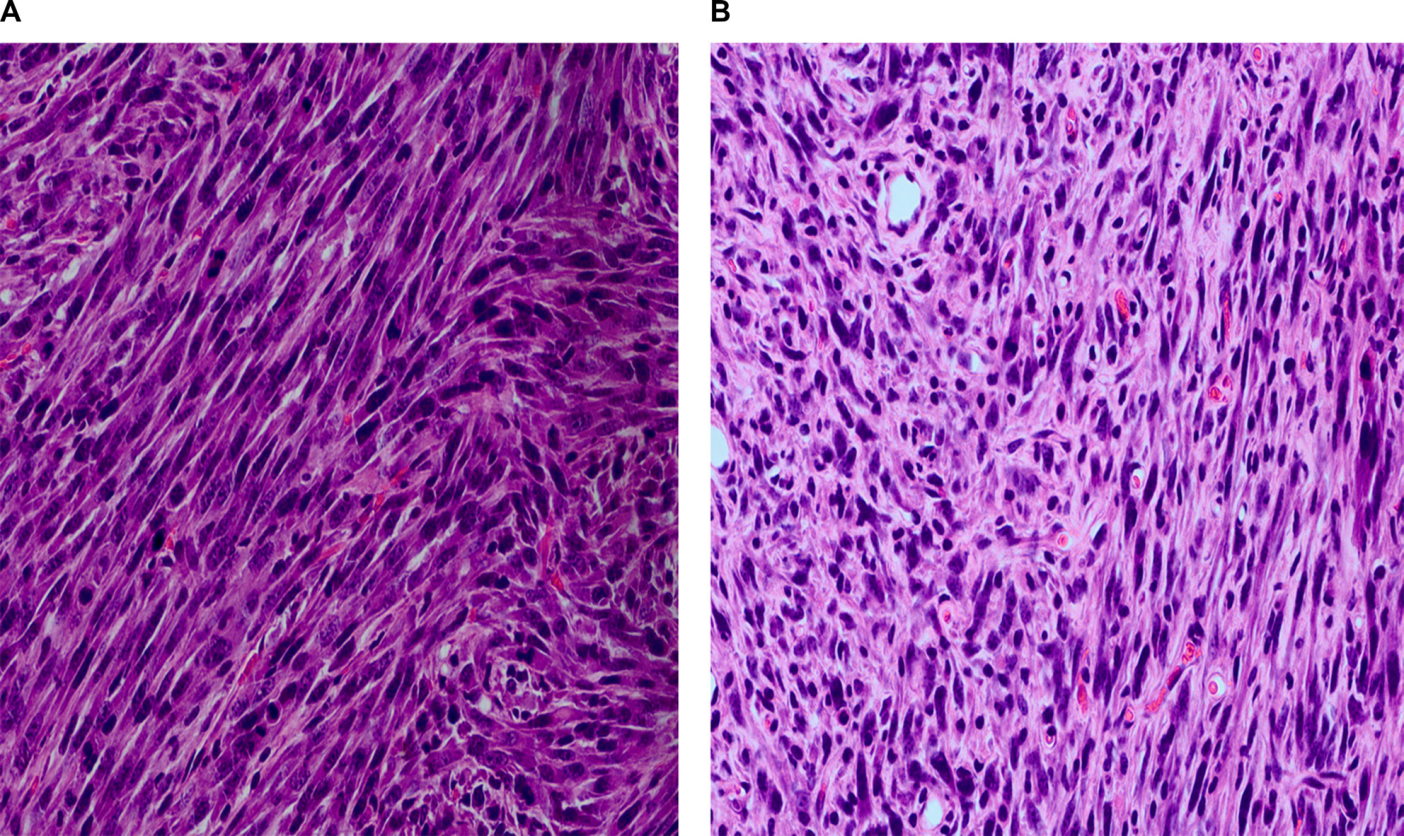
The HE staining of xenograft tumors of nude mice five weeks after subcutaneously injection of 5 × 10^6^ 3T3cells carrying KIF5B-RET fusion gene (**A**) Injection of 3T3 cells carrying KIF5B-RET-variant2 gene. (**B**) Injection of 3T3 cells carrying KIF5B-RET-variant4 gene.

In order to verify the malignant transformation ability of KIF5B-RET fusion gene, transformed and parental NIH3T3 cells (5 × 10^6^) were injected subcutaneously to 6-week-old female nude mice. Four weeks later, the tumors grew to a diameter of about 1cm in KV-2 and KV-4 groups, and the parental NIH3T3 cells had no tumorigenicity (data not shown). We then collected the xenograft tumors and conducted HE staining. Morphologically, tissues were similar to sarcoma tissue, and more mitotic figures and abnormal nuclei could be seen (Figure 2), confirming that KIF5B-RET fusion gene could induce the malignant transformation of fibroblast cell lines of 3T3.

### The functional role of KIF5B-RET fusion gene in cell proliferation, migration and invasion

**Figure 3:**
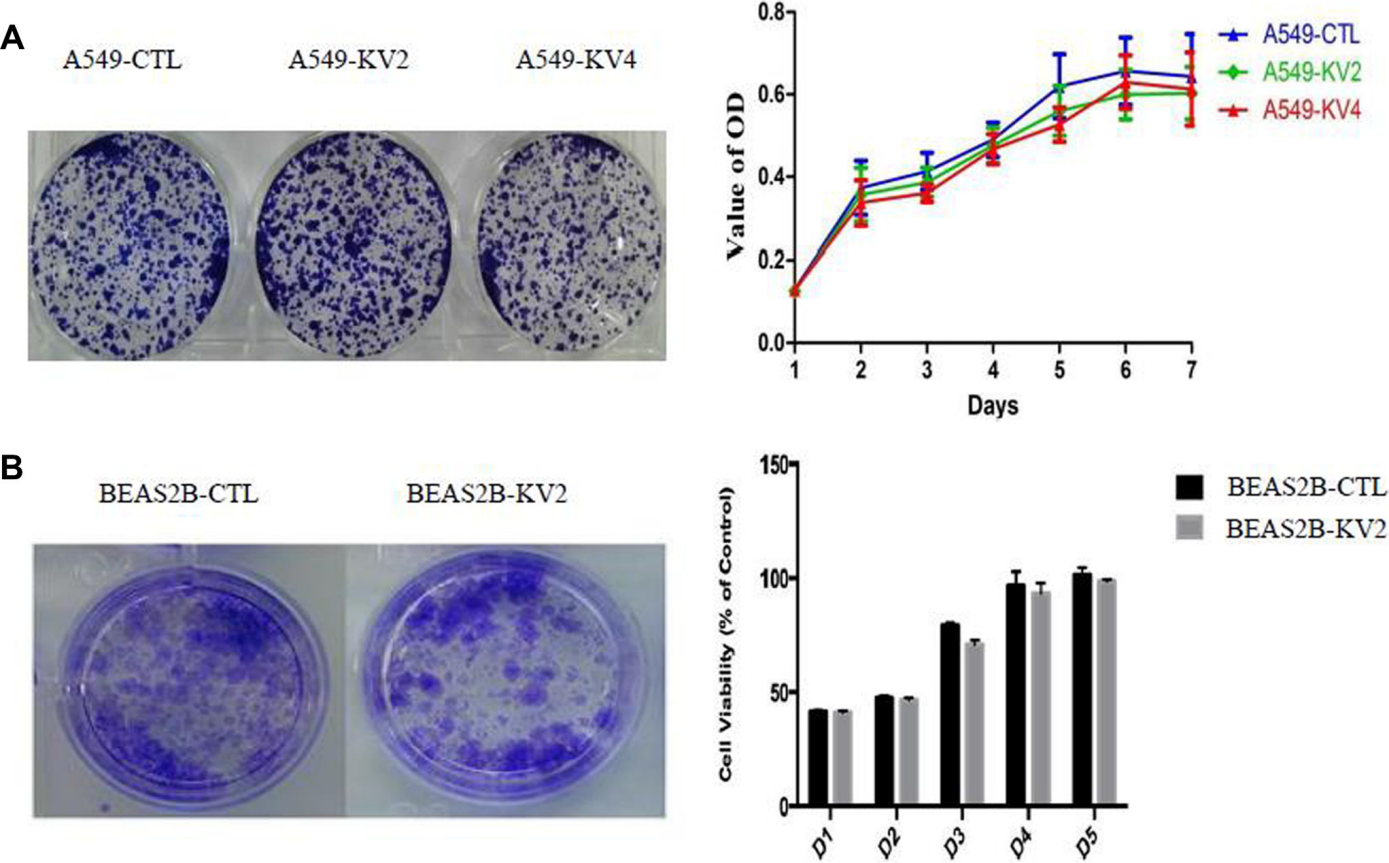
The proliferative and colony-forming abilities of KIF5B-RET transfected A549 and BEAS2B cells and the control groups (**A**). A549 cells. (**B**). BEAS2B cells. The result showed no significant difference in proliferation rate or colony number and size.

**Figure 4:**
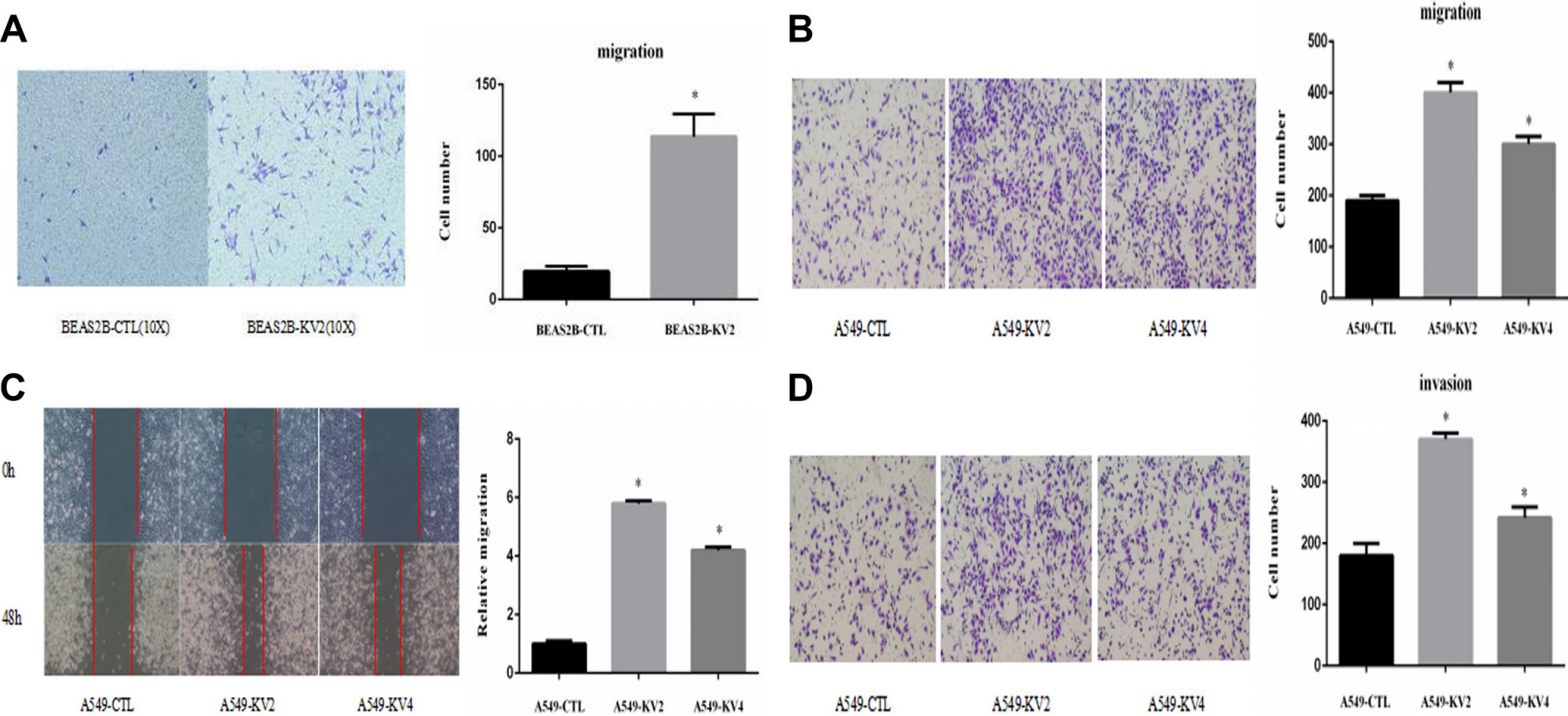
KIF5B-RET fusion gene promoted migration and invasion of cancer cells *in vitro* (**A**) Cell migration assay showed that KIF5B-RET promoted migration in BEAS2B-KV2 cells. (**B**) Cell migration assay showed that KIF5B-RET promoted migration in A549-KV2 and A549-KV4 cells. (**C**) Wound-healing assay showed that KIF5B-RET promoted migration in A549-KV2 and A549-KV4 cells. (**D**) Tumor invasion assay showed that KIF5B-RET promoted invasion in A549-KV2 and A549-KV4 cells. *P < 0.05.

We compared the cell proliferative and colony-forming abilities between KIF5B-RET transfected A549 and BEAS2B cells and the control groups, and found no significant difference in proliferation rate or colony number and size (Figure 3). The migration ability of KIF5B-RET transfected A549 cells and BEAS2B cells were detected by using transwell chambers. The results showed that more transfected cells intruded into bottom chamber than the negative control cells both in BEAS2B cells and A549 cells lines (Figure 4A, 4B). Wound-healing assay was used to evaluate the effect on A549 migration, as shown in Figure 4C, after 48 hours, A549-KV2 and A549-KV4 cells migrated significantly close to the scratched wound than negative control cells, displaying greater migration ability (Figure 4C). Tumor invasion assay, another characteristic contributing to cancer invasion and metastasis, was conducted in A549 cell line to assess invasiveness, and indicated that KIF5B-RET fusion gene could also promote invasion of A549 cells with KV2 or KV4 (Figure 4D).

### Signaling pathways involved in the migration and invasion of KIF5B-RET positive cells

**Figure 5:**
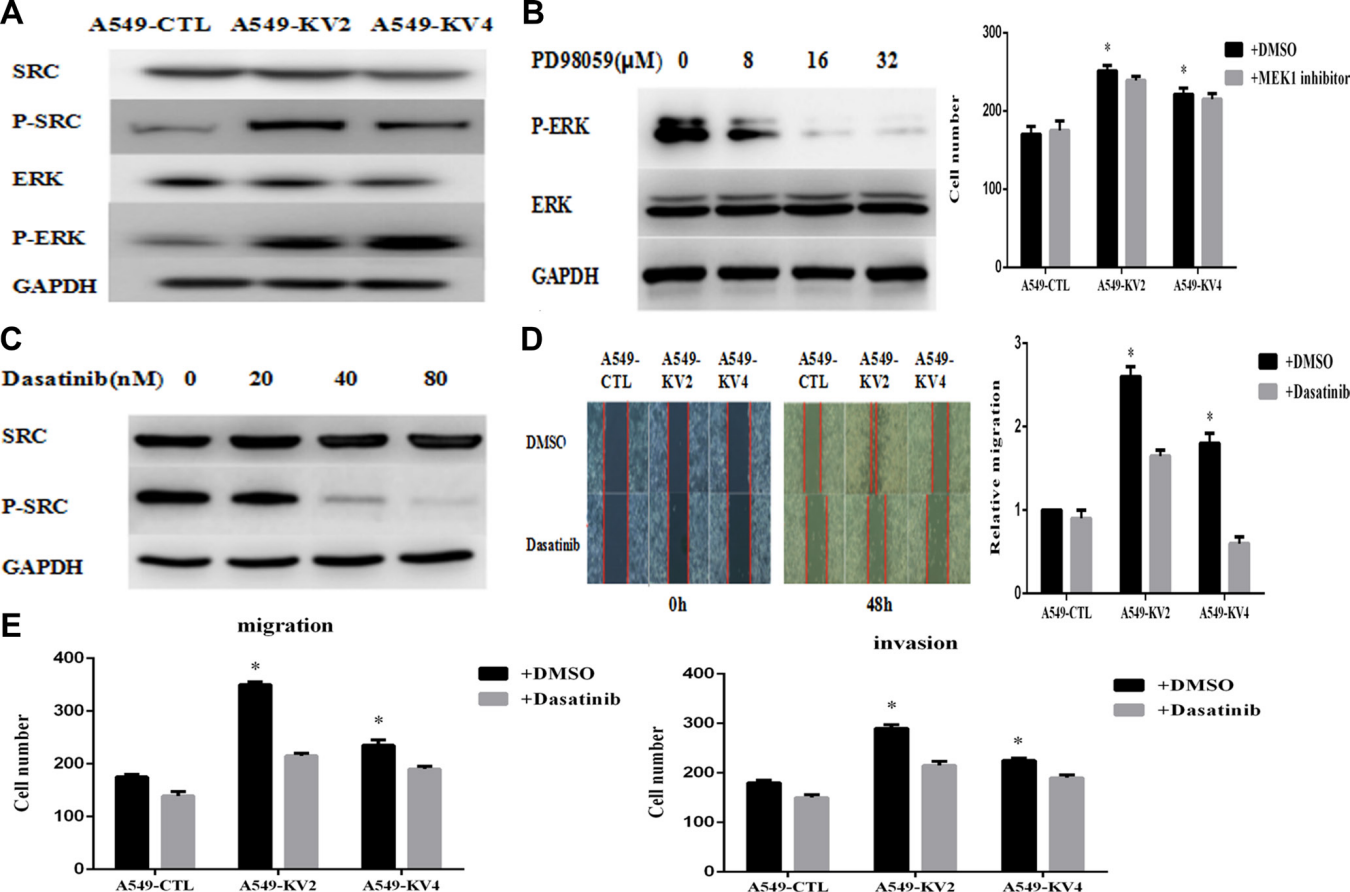
Signaling pathway involved in the migration and invasion of KIF5B-RET positive cells (**A**) p-Src and p-ERK were remarkably up-regulated in KIF5B-RET positive A549 cells. (**B**) p-ERK was totally inhibited by PD98059 at the concentration of 16 μM. On this condition, no significant difference was shown in migration ability between KIF5B-RET positive A549 cells and negative control cells. (**C**) p-Src was obviously inhibited by Dasatinib at the concentration of 40 nM. (**D**) Treated with Dasatinib at a concentration of 40 nM for 48 h, KIF5B-RET positive A549 cells hardly migrated in scratch assays, indicating the inhibiton of Dasatinib in migration. (**E**) Using Dasatinib with a concentration of 40 nM could significantly inhibited migration and invasion in KIF5B-RET positive A549 cells, indicating that Src protein may be the downstream pathway of KIF5B-RET to mediate invasion and migration. *P < 0.05.

Since KIF5B-RET fusion gene could promote migration and invasion of A549 cells, we tried to explore the downstream signaling pathways. The phosphorylation levels of migration and invasion-related signaling molecules were tested and we found that p-Src and p-ERK were remarkably up-regulated in KIF5B-RET positive A549 cells (Figure 5A). We then used a series of concentration of MEK1 inhibitor PD98059, which targeted the upstream signaling pathway of ERK1/2, and Src inhibitor Dasatinib to treat the cells. The concentration was ensured as 16 μM and 40 nM to obviously inhibit the p-ERK and p-Src(Figure 5B, 5C), and at this certain concentration, Dasatinib was observed to significantly reduce migration and invasion of KIF5B-RET positive A549 cells (Figure 5D, 5E), while PD98059 did not(Figure 5B), indicating that Src protein may be the downstream pathway of KIF5B-RET to mediate invasion and migration.

### Apatinib inhibited cell proliferation, migration and invasion of KIF5B-RET transfected A549 cells

**Figure 6:**
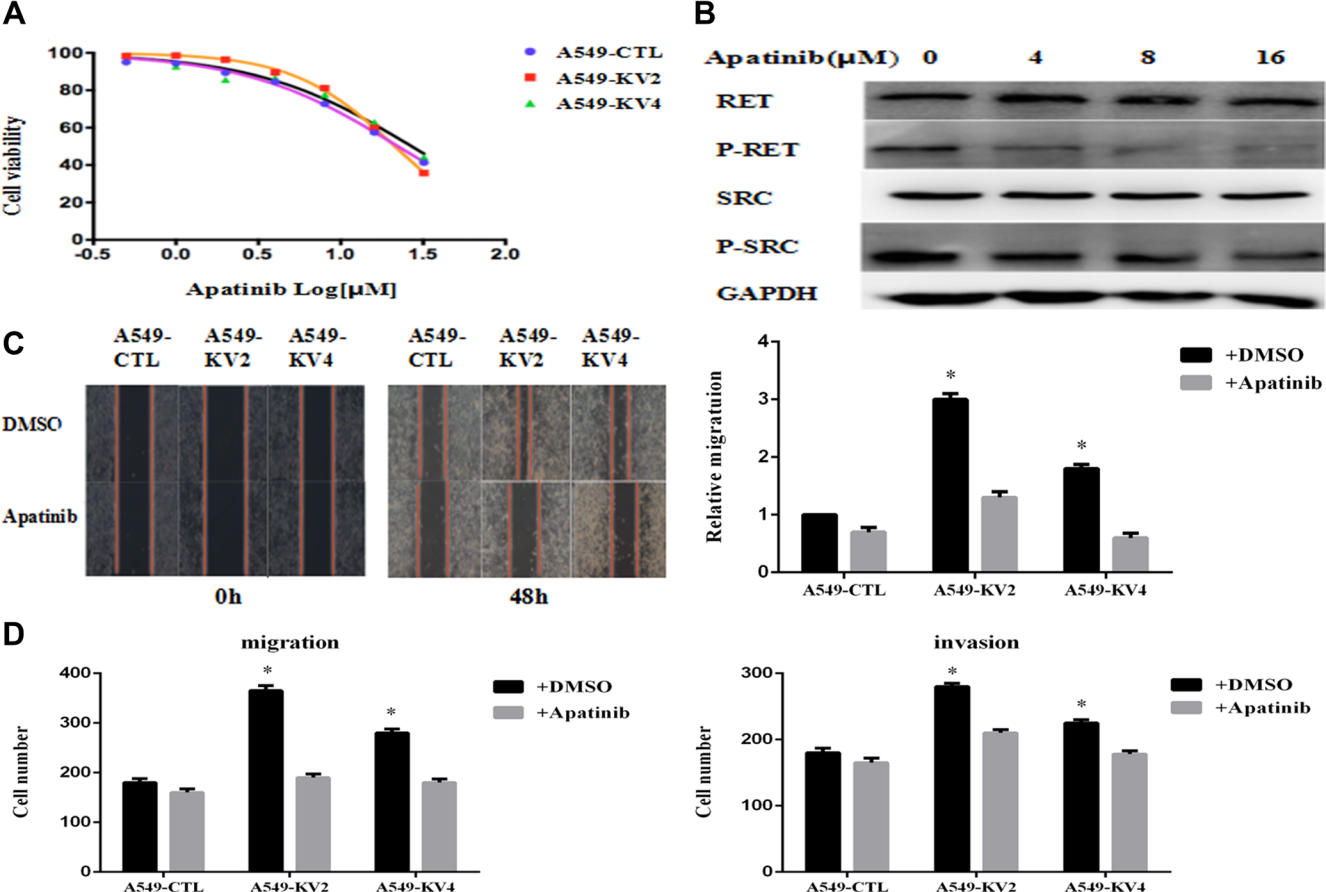
Apatinib inhibited cell proliferation, migration and invasion of KIF5B-RET transfected A549 cells (**A**) KIF5B-RET transfected A549 cells and the negative control cells were treated with Apatinib at different concentration for 48 h, and found similar suppressed cell viability in a concentration-dependent manner but without obvious difference in inhibition efficiency. (**B**) Western Blot showed the variation of protein expression after treating cells with concentration-gradient Apatinib, and found a dose-independent reduction of the phosphorylation of RET gene fusion kinases and the p-RET was totally inhibited at the concentration of 8 μM, along with the inhibition of p-Src. (**C**) Treated with Apatinib at a concentration of 8 μM for 48 h, KIF5B-RET positive A549 cells hardly migrated in scratch assays, indicating the inhibiton of Apatinib in migration. (**D**) Using Apatinib with a concentration of 8 μM could significantly inhibited migration and invasion in KIF5B-RET positive A549 cells. *P < 0.05.

We evaluated Apatinib's capacity to inhibit cell growth in KIF5B-RET transfected A549 cells and the control groups, and observed a suppressed viability in a concentration-dependent manner although there was no obvious difference between the inhibition efficiency of KIF5B-RET transfected cells and the control groups (Figure 6A). We then analyzed the variation of protein expression by Western Blot, and found Apatinib caused a dose-independent reduction of the phosphorylation of RET gene fusion kinases and the p-RET was totally inhibited at the concentration of 8 μM, along with the inhibition of p-Src (Figure 6B). On this condition, the migration and invasion effects were significantly reduced in KIF5B-RET driven A549 cells, indicating Apatinib may be a promising anti-metastatic agent to reduce migration and invasion (Figure 6C, 6D).

## DISCUSSION

In our study, we confirmed that KIF5B-RET expression in NIH3T3 cells could lead to oncogenic transformation, which was consistent with previous reports [7]. The functional characteristics of KIF5B-RET were mainly focused on the proliferative effect in published literature, but whether KIF5B-RET affect the metastasis of human NSCLC cells has never been investigated. Our results showed that RET overexpression significantly enhanced migration and invasion of KIF5B-RET positive A549 cells and Src protein might be involved in the signaling pathways to mediate invasion and migration. This phenomenon was in a way consistent with the clinical implications that RET-rearranged tumors were prone to be more prevalent in patients at advanced stage or with metastatic diseases [17–19]. This might facilitate the selection of patients for the screening of RET rearrangements and further treatment of RET inhibitors. However, we did not detect cell proliferation effect in KIF5B-RET transfected BEAS2B or A549 cells, which was different from the report of Qian et al., showing that KIF5B-RET fusion kinase promotes cell growth by multilevel activation of STAT3 in lung cancer [20].

Information about signal transduction downstream of the KIF5B-RET is still limited. Src, a membrane-associated nonreceptor tyrosine kinase, participates in the development of tumor metastasis through down-regulation of E-cadherin, up-regulation of matrix-degrading proteases (MMPs), activation of focal adhesion kinase (FAK), etc. [21–22]. In our experiment, compared with parental A549 cells, KIF5B-RET positive A549 cells had a stronger capability for migration and invasion, along with a remarkably up-regulation of p-Src. We then used Src inhibitor Dasatinib to treat the cells, and found migration and invasion ability of KIF5B-RET positive A549 cells was significantly reduced, indicating that Src protein is activated in the downstream pathway of KIF5B-RET driven tumors.

The biological functions of Apatinib in KIF5B-RET driven A549 cells were also investigated for the first time. In our study, we found Apatinib could inhibit the proliferation, migration and invasion in RET-rearranged LADC. But there was no obvious difference in inhibition efficiency of cell viability between KIF5B-RET transfected A549 cells and A549 cells. This might because the major effect of Apatinib in suppressing cell proliferation was performed through VEGF/VEGFR signaling pathway in the transfected or parental A549 cell line, rather than through anti-RET pathway. The migration and invasion effects were significantly reduced in KIF5B-RET driven A549 cells, indicating Apatinib may be a promising anti-metastatic agent to reduce migration and invasion. As a RTK inhibitor targeting RET and c-Src, Apatinib suppressed autophosphorylation of RET and Src protein simultaneously in a concentration-dependent manner, showing that Apatinib exhibits potent inhibitory activity against RET fusion–positive cells by suppressing RET/Src pathway.

To date, more explorations are on-going in RET inhibitors to find the therapeutic potential as targeted drugs tailored for RET fusion-positive patients [23–26]. Although RET-inhibiting TKIs provide new therapeutic potential in RET-associated tumors, the adverse side effects that are associated with VEGFR or EGFR inhibition should not be ignored [27]. As the first generation of oral antiangiogenesis drug created in China, Apatinib has been known for its simplicity, compliance, and less side effects [28]. Presently, clinical trials of Apatinib in advanced NSCLC are on-going based on the similar mechanism by inhibiting vascular endothelial growth factor receptors [29]. Given our result that Apatinib inhibits cellular invasion and migration by fusion kinase KIF5B-RET, it may be possible of using Apatinib in tumors harboring RET gene fusions, especially those with metastatic diseases, which needs further animal studies or clinical trials to confirm.

## MATERIALS AND METHODS

### Compounds and cell lines

Human bronchial epithelial cell BEAS-2B, human lung cancer cells A549, and the mouse fibroblast cell NIH3T3 were obtained from the Cell Bank of Type Culture Collection of Chinese Academy of Sciences (Shanghai, China). Cells were cultured in DMEM or RPMI1640 supplemented with 10% fetal bovine serum (FBS) at 37°C in a humidified atmosphere with 5% CO_2_. Apatinib, PD98059 and Dasatinib were purchased from Selleck Chemical (Houston, TX, USA) at an initial concentration of 10 mM, 20 mM and 10 mM (in 1 mL DMSO). Apatinib and PD98059 were directly used without dilution. Dasatinib was diluted to a final concentration of 50 μM. The final DMSO concentration in cell treatment was 0.04%–0.16%.

### Lentivirus production and transduction

Plasmids that express KIF5B-RET Variant2, KIF5B-RET Variant4 were kindly gifted from Department of Thoracic Surgery, Fudan University Shanghai Cancer Center. Lentiviral vector system (Tronolab) was a four plasmid system, which was composed of pRsv-REV, pMDlg-pRRE, pMD2G, and target interference plasmid expressing green fluorescent protein (GFP). Cell line 293T, lentivirus packaging cell, was cultured in DMEM containing 10% FBS. E. coli strain DH5α was used to amplify lentiviral vectors and help package vector plasmids. Stable transfected A549, Beas-2B and 3T3 cells were constructed by infection of KIF5B-RET-FLAG-expressing lentiviruses.

### Cell proliferation assays

BEAS-2B and A549 cells were seeded at a density of 2000 cells and 500 per well in 96-well plates. The cells were transfected with KIF5B-RET Variant2 or KIF5B-RET Variant4. Cell proliferation was analyzed using Cell Counting Kit 8 (Dojindo, Kumamoto, Japan) according to the manufacturer's protocol.

### Colony formation assays

A549 cells were seeded into 6-well plates at a concentration of 5000 cells per well. Cells were cultured for 10 days. At the end of incubation, cells were fixed with methanol for 10 min and stained with crystal violet for 10∼15 min.

### Scratch assays

Cells were seeded in a 6-well plate, and a “wounding” line was scratched in the center of the cell monolayers with a sterile 200-μL pipette tip. The debris was removed by washing at least twice with PBS. Serum - free medium were added. The width of the wound was measured under a microscope at 0 and 48 hours after the scratch to assess the migration ability of the cells.

### Cell migration and invasion assays

Migration and invasion experiments *in vitro* were carried out in the chamber of 8-μm transwell inserts (BD Falcon™; Becton Dickinson, Franklin Lakes, NJ, USA) with or without Matrigel (BD Falcon™). An amount of 10^5^ A549 cells were incubated in serum-free medium at the top chamber of each well insert, and serum-containing medium were added to the lower chamber. Cells that migrated were fixed in 10% formalin and stained with 1% crystal violet after 24 hours of incubation at 37°C, and were counted under a light microscope at a magnification of ×200.

### Western blot

Total protein lysates were obtained from cultured cells using radio-immunoprecipitation assay buffer supplemented with complete protease inhibitor cocktail (Roche, Basel, Switzerland). Protein concentrations were determined using the BCA protein assay kit (Biyotime, Shanghai, China). Cell extracts were subjected to sodium dodecyl sulfatepolyacrylamide gel electrophoresis and were transferred into polyvinylidene fluoride (PVDF). The membrane was then blocked with 5% skim milk in TBST for 2 h at room temperature and probed with the primary antibodies overnight at 4°C. After washing with TBST, the membrane was incubated with horseradish peroxidase (HRP)–conjugated secondary antibody for 1 h at room temperature, washed three times with TBST, and detected by enhanced chemiluminescence reagent (Pierce, Rockford, IL, USA). Antibodies against RET, p-RET, ERK, p-ERK, Src, p-Src, GAPDH, β-actin were purchased from Cell Signaling Technology (Cambridge, MA, USA).

### Xenograft tumor model and hematoxylin-eosin staining

5 × 10^6^ NIH3T3 cells carrying KIF5B-RET Variant 2, KIF5B-RET Variant 4 and the control NIH3T3 cells were injected subcutaneously to 6-week-old female nude mice. The tumors were measured every week until 1 month later. The xenograft tumor samples were fixed in 10% neutral-buffered formalin for 24 h and embedded in paraffin. Sections were reacted with hemalum for nuclear staining, and then counterstained with eosin for staining of other eosinophilic structures.

### Statistical analysis

All experiments were repeated at least thrice. Statistical analysis was performed using Statistical Package for the Social Sciences (SPSS) software version 22.0 for Windows (SPSS Inc., Chicago, USA). Differences between groups were calculated by using the Student's *t*-test. The statistical significance was determined at *P*-value < 0.05. Graphs were created with GraphPad Prism 5.

## Abbreviations

NSCLC: Non-Small Cell Lung Cancer
RET: Rearranged during Transfection
KIF5B: Kinesin Family 5B
LADC: Lung Adenocarcinomas
TKIs: Tyrosine Kinase Inhibitors
RTK: Receptor Tyrosine Kinase
VEGFR-2: Vascular Endothelial Growth Factor Receptor-2
PDGFR-β: Platelet-Derived Growth Factor-β
c-Src-v: Src Sarcoma Viral Oncogene Homolog
c-Kit: Stem Cell Factor Receptor
FBS: Fetal Bovine Serum
GFP: Green Fluorescent Protein
PVDF: Polyvinylidene Fluoride
HRP: Horseradish Peroxidase
SPSS: Statistical Package for the Social Sciences
MMPs: Matrix Metalloproteninases
FAK: Focal Adhesion Kinase.
